# Evaluation of Plasma-Derived hsa_circ_003077 for Non-Invasive Diagnosis of Alzheimer’s Disease

**DOI:** 10.3390/biom16030356

**Published:** 2026-02-26

**Authors:** Hamit Çelik, Oğuz Çelik, Şeyma Aydın, Sefa Küçükler, Selim Çomaklı, Ramazan Akay, Sinan Gönüllü, Mustafa Onur Yıldız, Bülent Alım, Selçuk Özdemir

**Affiliations:** 1 Department of Neurology, Private Buhara Hospital, 25240 Erzurum, Türkiye; 2 Savur Prof. Dr. Aziz Sancar District State Hospital, 47860 Mardin, Türkiye; 3 Department of Genetics, Faculty of Veterinary Medicine, Atatürk University, 25240 Erzurum, Türkiye; 4 Department of Biochemistry, Faculty of Veterinary Medicine, Atatürk University, 25240 Erzurum, Türkiye; 5 Department of Pathology, Faculty of Veterinary Medicine, Atatürk University, 25240 Erzurum, Türkiye; 6 Department of Neurology, Eskisehir City Hospital, 26080 Eskişehir, Türkiye; 7 Department of Neurology, Bursa City Hospital, 16250 Bursa, Türkiye; 8 Department of Neurology, Faculty of Medicine, Samsun University, 55060 Samsun, Türkiye

**Keywords:** Alzheimer’s disease, circular RNA, biomarkers, receiver operating characteristic, TAM receptors

## Abstract

Alzheimer’s disease (AD) is a progressive neurodegenerative disorder affecting the central nervous system and is the most common form of dementia in the elderly. Current diagnostic methods are limited in the early and definitive diagnosis of the disease, necessitating the need for new and more reliable biomarkers. Circular RNAs (circRNAs) are non-coding, single-stranded, and highly stable RNA molecules commonly found in the eukaryotic transcriptome. Recent studies have shown that changes in the expression levels of circRNAs may play a role in AD pathogenesis. Furthermore, these molecules are considered as potential non-invasive biomarkers for early diagnosis of AD. In this study, we comprehensively assessed plasma levels of classical neurodegenerative biomarkers [amyloid-β42/amyloid-β40 (Aβ42/Aβ40) ratio, total Tau (tTau), and phosphorylated Tau (pTau)], as well as glial and inflammatory mediators, TAM receptor family members (Tyro3 and AXL), and the newly identified circular RNA molecule hsa_circ_003077. The findings revealed that the expression levels of TAM receptors were significantly increased, the Aβ42/Aβ40 ratio decreased, and both total Tau and phosphorylated Tau levels were significantly increased in AD patients. In the receiver operating characteristic (ROC) curve analysis performed to determine the diagnostic potential of hsa_circ_003077, the area under the curve (AUC) was 0.90 (95% CI: 0.82–0.97). This high AUC value suggests that hsa_circ_003077 may be a strong and novel biomarker candidate for the non-invasive diagnosis of AD. The data obtained confirmed the diagnostic efficacy of classical AD biomarkers and revealed that hsa_circ_003077 is a promising biomarker for early and accurate detection of the disease. However, in order to assess the transferability of these findings to clinical practice, confirmatory studies with larger sample groups are needed to ensure reproducibility of the results.

## 1. Introduction

With the aging of the global population, neurodegenerative diseases have emerged as a major public health challenge worldwide [1]. Among these disorders, Alzheimer’s disease (AD) is a progressive neurodegenerative condition with a complex etiology and represents the leading cause of dementia globally [2]. Histopathologically, AD is characterized by the extracellular accumulation of amyloid-β (Aβ) plaques and the intracellular formation of neurofibrillary tangles composed of aberrantly phosphorylated tau protein [3,4]. Importantly, these neuropathological alterations begin many years before the onset of clinical symptoms, underscoring the urgent need for early and accurate diagnostic strategies.

According to clinical guidelines published in 2011 by the National Institute on Aging and the Alzheimer’s Association, the diagnosis of AD-related dementia and mild cognitive impairment (MCI) relies primarily on clinical assessment, while cerebrospinal fluid (CSF) biomarkers and neuroimaging techniques such as magnetic resonance imaging (MRI) and positron emission tomography (PET) are mainly recommended to improve diagnostic confidence or to identify preclinical disease stages [5,6]. More recently, updated research criteria released by the Alzheimer’s Association in 2024 have proposed a biomarker-based biological definition of AD, suggesting that abnormalities in any Core 1 biomarker, including amyloid PET, approved CSF biomarkers, or validated plasma biomarkers, may support an AD diagnosis [7]. Nevertheless, these approaches remain limited by invasiveness, high cost, limited accessibility, and suboptimal sensitivity for early-stage detection [8]. Consequently, there is a growing interest in identifying reliable and minimally invasive biomarkers in readily accessible biofluids such as blood [9].

Biomarkers are defined by the FDA/NIH Biomarker Working Group as measurable biological characteristics that reflect normal biological processes, pathogenic mechanisms, or responses to therapeutic interventions [10]. In Alzheimer’s disease, biomarkers play a central role throughout the disease continuum, from early diagnosis and risk stratification to disease monitoring and prognostic evaluation [11]. Molecular biomarkers encompass a broad range of biological entities, including proteins, genetic variants, and non-coding RNAs (ncRNAs), such as microRNAs (miRNAs), long non-coding RNAs (lncRNAs), and circular RNAs (circRNAs) [12]. Increasing evidence indicates that ncRNAs, particularly circRNAs, are involved in regulatory networks relevant to AD pathophysiology, including neuroinflammation, synaptic dysfunction, and neuronal survival [13,14].

CircRNAs are a distinct class of endogenous ncRNAs generated through back-splicing of precursor mRNA transcripts, resulting in covalently closed circular molecules lacking free 5′ and 3′ ends [15,16]. This unique structure confers resistance to exonuclease-mediated degradation, leading to enhanced molecular stability compared with linear RNAs [17]. Functionally, circRNAs can act as competitive endogenous RNAs by sequestering miRNAs or interact with RNA-binding proteins to regulate post-transcriptional gene expression [18,19]. In addition, several studies suggest that certain circRNAs may exhibit altered expression at early stages of neurodegenerative processes, supporting their investigation as potential biomarkers for early disease detection [20]. Despite these theoretical advantages, the translational applicability of circulating circRNAs remains at an exploratory stage. Plasma circRNA detection is influenced by multiple biological and technical factors, including inter-individual variability, differences in cellular origin, RNA isolation efficiency, normalization strategies, and pre-analytical sample handling conditions [21,22,23]. Moreover, the relationship between circRNA expression in peripheral blood and central nervous system pathology is not yet fully understood, which may contribute to variability across studies. Recent reviews highlight that although circRNAs are abundant in brain tissues and detectable in peripheral biofluids, the mechanistic link between circulating circRNA levels and CNS disease processes remains to be established, particularly in neurodegenerative contexts such as AD [24,25]. Accordingly, claims regarding the immediate clinical utility of plasma circRNAs should be interpreted with caution, and further validation in large, well-characterized cohorts is required.

Recent profiling studies have identified multiple differentially expressed circRNAs in the brain tissue, CSF, and peripheral blood of patients with Alzheimer’s disease, highlighting their potential relevance as disease-associated molecular signatures [26,27,28]. In a comprehensive microarray-based study, Li et al. [26] reported significant dysregulation of circRNAs in AD, with 112 circRNAs upregulated and 51 circRNAs downregulated compared with control subjects. Among these, hsa_circ_003077 was found to be significantly downregulated in the CSF of AD patients and showed a negative correlation with disease risk and cognitive impairment severity. Notably, this circRNA was consistently detected across patient samples and exhibited a robust association with clinical parameters, providing a rationale for its prioritization among the dysregulated candidates. Building upon these observations, the present study explores hsa_circ_003077 expression in plasma, a less invasive and more readily accessible biofluid. This investigation is designed as a hypothesis-driven exploratory assessment aimed at evaluating the detectability and discriminatory potential of hsa_circ_003077 in peripheral blood, rather than asserting immediate clinical applicability.

## 2. Materials and Methods

### 2.1. Cohort Information

This study included participants from two clinical categories: cognitively healthy individuals (HC) and patients diagnosed with Alzheimer’s disease (AD) dementia. Participants were recruited according to established inclusion and exclusion criteria based on standard clinical diagnostic guidelines. All participants were enrolled at Erzurum Buhara Private Hospital, Neurology Clinic, following standardized procedures (Table 1). In accordance with the Declaration of Helsinki, written informed consent was obtained from each participant or their legal representatives. The study protocol was approved by the Ethics Committee of Atatürk University Faculty of Medicine, Clinical Research Ethics Committee (Approval number: B.30.2.ATA.0.01.00/5069).

### 2.2. Pre-Analytical Management

Peripheral blood samples were obtained in tubes containing EDTA, which were then kept on ice and processed within two hours of collection. Plasma was obtained by centrifuging the samples at 2000× *g* for 15 min at 4 °C. The plasma samples obtained were aliquoted and preserved at −80 °C. To maintain the structural and functional integrity of the biomarkers, repeated freeze-thaw cycles were avoided.

### 2.3. Calibration and Quality Assurance

All biomarker analyses were performed according to the protocols recommended by the manufacturers and using appropriate calibration curves and quality control (QC) samples. Standard curves were generated by running calibration standards on each assay plate. Internal quality control samples representing different concentration levels (high, medium and low) were used to evaluate assay performance. The coefficient of variation (CV) of the quality control samples was less than 15%, indicating an acceptable level of reliability of the assay results.

### 2.4. Biomarker Measurement

Aβ40, Aβ42, total tau (tTau), and phosphorylated tau at threonine 181 (pTau181) concentrations were quantitatively determined using the V-PLEX Neurodegeneration Panel developed by Meso Scale Discovery (MSD, Rockville, MD, USA). Measurements were performed on MSD QuickPlex SQ 120 or Sector S 600 platforms using the electrochemiluminescence (ECL) method, which allows multi-analyte detection. Plasma samples were thawed on ice prior to analysis and diluted 1:2 ratio according to the manufacturer’s specifications. The limit of detection (LOD) for analytical performance parameters was defined as ~5 pg/mL for Aβ40, ~2 pg/mL for Aβ42, ~0.05 pg/mL for tTau, and ~0.1 pg/mL for pTau181. The lower limit of quantification (LLOQ) was defined as ~10 pg/mL for Aβ40, ~5 pg/mL for Aβ42, ~0.1 pg/mL for tTau, and ~0.2 pg/mL for pTau181.

Phosphorylated tau 217 (pTau217) levels in plasma were quantitatively measured on the Simoa SR-X analyzer using the Quanterix Simoa^®^ pTau-217 Advantage Kit (Quanterix, Billerica, MA, USA). This experiment utilized Single Molecule Array (Simoa) technology for ultrasensitive measurement of plasma pTau217.

For analytical performance characteristics, the LOD for pTau217 was approximately 0.06 pg/mL and the LLOQ was approximately 0.12 pg/mL. Plasma samples were processed undiluted or with minimal dilution according to the protocol provided by the manufacturer, and each sample was analyzed in duplicate.

The levels of Neurofilament Light Chain (NfL) and Glial Fibrillary Acidic Protein (GFAP) were measured simultaneously using the MSD V-PLEX Neurodegeneration Biomarker Panel 1 (Meso Scale Discovery, Rockville, MD, USA). Analyses were performed on the MSD QuickPlex platform based on the ECL detection method.

Analytical characteristics, the LOD for NfL was approximately 3 pg/mL, and for GFAP, it was approximately 1 pg/mL. The LLOQ was approximately 10 pg/mL for NfL and approximately 5 pg/mL for GFAP, respectively. All plasma samples were processed at a 1:4 dilution before analysis.

Enzyme-linked immunosorbent assay (ELISA) was used to measure human AXL and Tyro3 levels in plasma according to the manufacturer’s protocols. The Human Tyro3 DuoSet ELISA kit (Catalog No: DY8596-05) supplied by R&D Systems (Minneapolis, MN, USA) was used to measure Tyro3 levels, while the Human AXL DuoSet ELISA kit (Catalog No: DAXL00; R&D Systems, Minneapolis, MN, USA) was used to measure AXL levels. All samples were analyzed in duplicate, and standard curves were generated using recombinant protein standards supplied with the kit. The concentrations of the samples were calculated based on the standard curves obtained with a four-parameter logistic (4-PL) regression model implemented in R (v4.1.0, R Foundation for Statistical Computing, Vienna, Austria).

The analytical performance parameters of the ELISA kits used for the quantitative determination of AXL and Tyro3 levels were evaluated according to the data provided by the manufacturer. For AXL, the measurement range was 62.5–4000 pg/mL, the LOD was 18 pg/mL, and the LLOQ was 62.5 pg/mL. For Tyro3, the measurement range was 31.2–2000 pg/mL, LOD was 10 pg/mL, and LLOQ was 31.2 pg/mL. To ensure the accuracy and reliability of the measurements, intra-assay and inter-assay coefficients of variation (%CV) were kept below 10%.

### 2.5. RT-PCR

Total RNA was isolated from plasma samples using the QIAamp RNA Blood Mini Kit (Qiagen, Hilden, Germany) according to the manufacturer’s instructions. RNA concentration and purity were assessed spectrophotometrically using a NanoDrop instrument (Thermo Fisher Scientific, Waltham, MA, USA) by measuring absorbance ratios at 260/280 nm.

Complementary DNA (cDNA) was synthesized from plasma RNA using the SuperScript IV VILO Master Mix (Thermo Fisher Scientific, Waltham, MA, USA) following the manufacturer’s protocol. Quantitative real-time PCR (RT-qPCR) was performed using the Rotor-Gene Q real-time PCR system (Qiagen, Hilden, Germany) to assess hsa_circ_003077 expression using divergent primers spanning the back-splice junction (forward: 5′-GTGGAAGTTGATGGGTCGA-3′; reverse: 5′-GTGACATGGTTCTTTGACTTACGA-3′). PCR amplification was carried out with an initial denaturation at 95 °C for 10 min, followed by 40 cycles of denaturation at 95 °C for 15 s and annealing/extension at 60 °C for 1 min.

Relative expression levels of hsa_circ_003077 were quantified using the 2^−ΔCt method with normalization to GAPDH. All reactions were performed in triplicate, and no-template controls were included to exclude contamination.

Genomic annotation of circ_0030777, including host gene information, chromosomal location, and reference database identifiers (circBase and circAtlas), is provided in Supplementary Table S1.

### 2.6. Statistical Analysis

All statistical analyses were performed using R (v4.1.0, R Foundation for Statistical Computing, Vienna, Austria) software. Statistical significance was considered for *p*-values < 0.05. The normality of the data was assessed using the Shapiro–Wilk test, and it was determined that the data were non-parametric, with significant deviations from normal distribution in most cases. Consequently, non-parametric tests were chosen for group comparisons. Biomarker levels between cognitively healthy controls (HC) and Alzheimer’s disease (AD) patients were compared using the Mann–Whitney U test, which is appropriate for independent groups when data do not meet normality assumptions.

Receiver operating characteristic (ROC) curve analysis was performed using R software, and ROC curves were generated to assess the diagnostic accuracy of biomarkers in distinguishing Alzheimer’s disease from cognitively healthy controls. The area under the ROC curve (AUC) was calculated, and the optimal cutoff value for each biomarker was determined using the Youden index to maximize sensitivity and specificity. AUC values were categorized as AUC 0.90–1.00: Superior diagnostic efficacy, AUC 0.80–0.89: Good diagnostic efficacy, AUC 0.70–0.79: Moderate diagnostic efficacy, and AUC < 0.70: Low diagnostic efficacy.

Spearman’s rank correlation analysis was performed using R software to examine the correlations between biomarker levels. Spearman correlation coefficient (ρ) was calculated to evaluate the strength and direction of monotonic correlations. The significance of the correlation was assessed using the two-tailed test, and *p*-values < 0.05 were considered statistically significant. The correlation matrix was visualized with a heatmap for better understanding the relationships between biomarkers.

Given the exploratory nature of the study, no adjustment for multiple testing was applied to the correlation analyses; therefore, the reported associations are intended to be hypothesis-generating rather than confirmatory.

## 3. Results

### 3.1. Plasma Biomarker Profiles and hsa_circ_003077 Expression Between AD Patients and Healthy Controls

**Figure 1:**
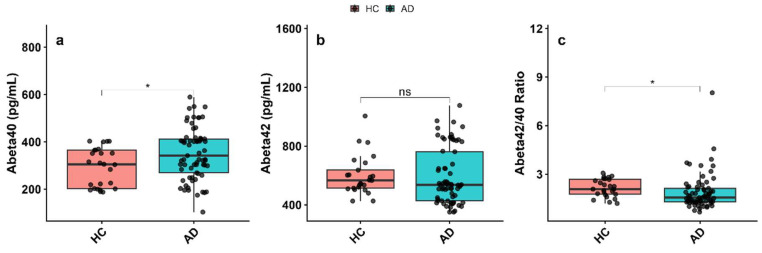
Plasma Aβ levels in Alzheimer’s disease (AD) patients and cognitively healthy controls (HC). (**a**) Aβ40, (**b**) Aβ42, and (**c**) Aβ42/Aβ40 ratio were measured in plasma samples. Data are presented as mean ± SEM. Statistical comparisons between groups were performed using the Mann–Whitney U test. Aβ40 levels were significantly increased in AD patients, whereas Aβ42 levels showed no significant difference; however, the Aβ42/Aβ40 ratio was significantly decreased in the AD group. Statistical significance is indicated as * *p* < 0.05; ns, not significant.

Plasma biomarker analysis revealed significant alterations between the AD and HC groups. Aβ40 levels were significantly increased in the AD group compared to the HC group (*p* < 0.05, Figure 1a). However, Aβ42 levels did not differ significantly between the groups (*p* > 0.05, Figure 1b). In contrast, the Aβ42/Aβ40 ratio was significantly decreased in the AD group (*p* < 0.05, Figure 1c).

**Figure 2:**
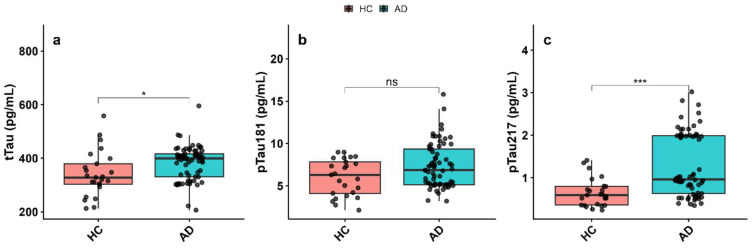
Plasma tau levels in Alzheimer’s disease (AD) patients and cognitively healthy controls (HC). (**a**) Total tau (tTau), (**b**) phosphorylated tau-181 (pTau-181), and (**c**) phosphorylated tau-217 (pTau-217) levels were measured in plasma. Data are shown as mean ± SEM. Statistical comparisons were performed using the Mann–Whitney U test. Statistical significance is indicated as * *p* < 0.05; *** *p* < 0.001; ns, not significant.

In contrast, levels of total tau (tTau) (Figure 2a) and phosphorylated tau-217 (pTau-217) (Figure 2c) were significantly elevated in AD patients compared with the HC group (tTau: *p* < 0.05; pTau-217: *p* < 0.001; Supplementary Table S2). However, phosphorylated tau-181 (pTau-181) levels showed no significant difference between AD and HC groups (Figure 2b, *p* > 0.05). These findings indicate the presence of tau-related pathology in the AD group and support the utility of plasma tau biomarkers—particularly pTau-217—in differentiating AD from healthy aging.

**Figure 3:**
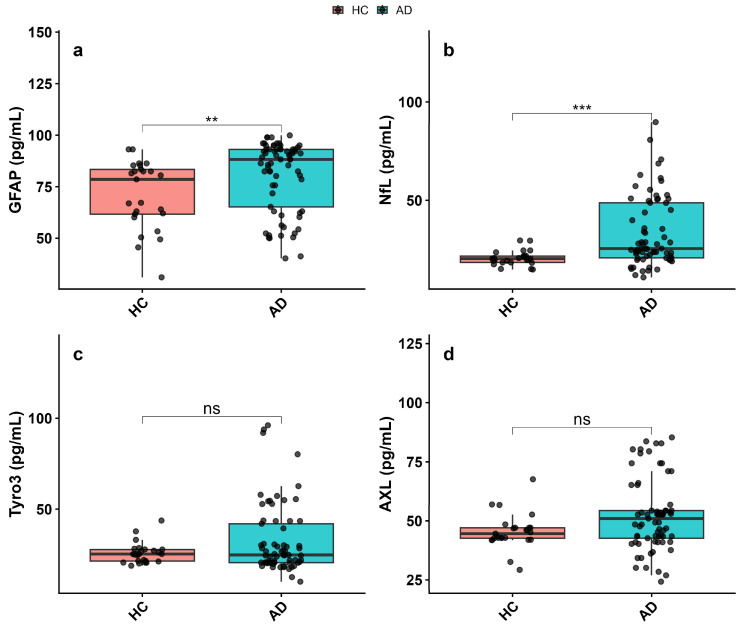
Plasma glial and receptor biomarkers in Alzheimer’s disease (AD) patients and cognitively healthy controls (HC). (**a**) GFAP, (**b**) NfL, (**c**) Tyro3, and (**d**) AXL levels were measured in plasma. Data are presented as mean ± SEM. Statistical comparisons were performed using the Mann–Whitney U test. Statistical significance is indicated as ** *p* < 0.01; *** *p* < 0.001; ns, not significant.

GFAP levels were increased in AD patients compared to cognitively healthy controls, and this elevation reached statistical significance (*p* < 0.01, Figure 3a). NfL levels, a key marker of axonal damage, were also significantly higher in the AD group (*p* < 0.001, Figure 3b). In contrast, plasma Tyro3 and AXL levels showed no significant differences between AD patients and healthy controls (Tyro3: *p* > 0.05, Figure 3c; AXL: *p* > 0.05, Figure 3d) (Supplementary Table S2).

**Figure 4:**
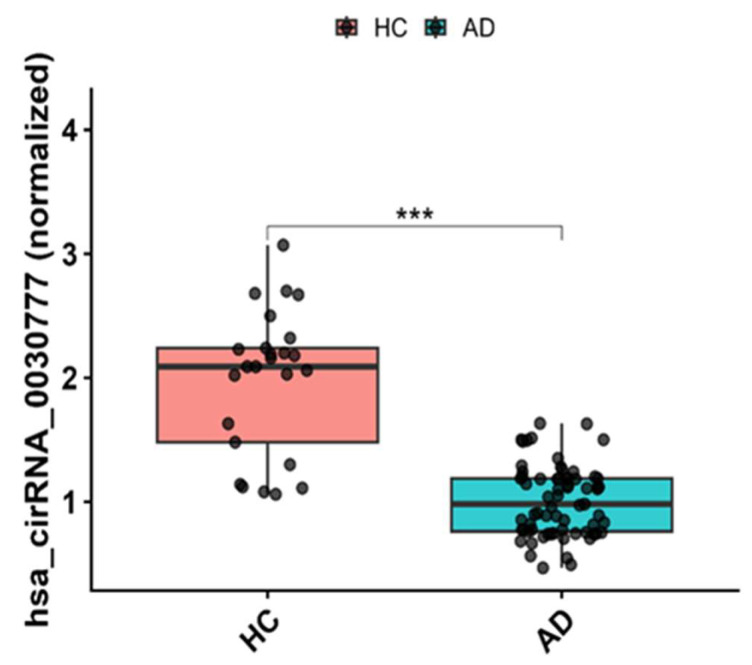
Plasma hsa_circ_003077 levels in Alzheimer’s disease (AD) patients and cognitively healthy controls (HC). Relative expression of hsa_circ_003077 was measured in plasma samples. Data are shown as mean ± SEM. Statistical comparisons were performed using the Mann–Whitney U test. *** *p* < 0.001 indicates a significant decrease in AD patients compared to HC.

When comparing circulating hsa_circ_003077 levels between AD patients and HC, a significant decrease was observed in the AD group (Figure 4). According to the graphical analysis, the relative expression of hsa_circ_003077 was markedly lower in the AD group, and this difference was statistically significant (*p* < 0.001) (Supplementary Table S2).

### 3.2. Evaluation of Biomarker Diagnostic Accuracy via ROC Analysis

**Figure 5:**
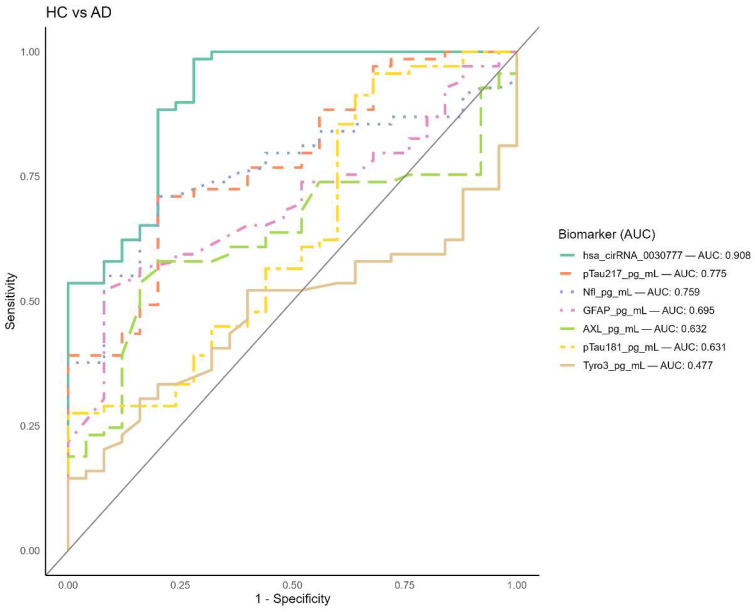
Diagnostic performance of plasma biomarkers for differentiating Alzheimer’s disease (AD) from cognitively healthy controls (HC) based on ROC curve analysis. Receiver operating characteristic (ROC) curves were generated to assess the diagnostic accuracy of plasma biomarkers. The area under the ROC curve (AUC) was used to evaluate biomarker performance: 0.90–1.00, superior diagnostic efficacy; 0.80–0.89, good diagnostic efficacy; 0.70–0.79, moderate diagnostic efficacy; <0.70, poor/substandard diagnostic efficacy. Data are presented as mean ± SEM. Statistical comparisons were performed using the Mann–Whitney U test. hsa_circ_003077 exhibited the highest diagnostic accuracy (AUC = 0.90; 95% CI: 0.82–0.97), followed by NfL (AUC = 0.75) and pTau-217 (AUC = 0.77). AXL and GFAP showed lower diagnostic performance (AUC = 0.63 and 0.69, respectively), while pTau-181 and Tyro3 demonstrated substandard efficacy (AUC = 0.63 each).

To evaluate the diagnostic precision of the biomarkers in distinguishing Alzheimer’s disease (AD) patients from healthy controls (HC), a ROC analysis was conducted (Figure 5). The area under the ROC curve (AUC) values were interpreted as follows: 0.90–1.00 indicates superior diagnostic efficacy, 0.80–0.89 denotes good diagnostic efficacy, 0.70–0.79 reflects moderate diagnostic efficacy, and values below 0.70 are considered to have poor or substandard diagnostic performance. According to the results, hsa_circ_003077 demonstrated the highest sensitivity and specificity, with an AUC of 0.90 (95% CI: 0.82–0.97, Supplementary Table S3), indicating it is a highly effective biomarker for differentiating AD from HC. pTau217 (AUC = 0.77) and NfL (AUC = 0.75) showed moderate diagnostic efficacy, while GFAP (AUC = 0.69) and AXL (AUC = 0.63) exhibited poor-to-borderline diagnostic performance. pTau181 and Tyro3 also displayed substandard diagnostic efficacy (AUC = 0.63 for each), suggesting limited utility in distinguishing AD from HC (Supplementary Table S4).

### 3.3. Correlation Analysis of Neurodegenerative and Inflammatory Biomarkers in AD and Healthy Controls

**Figure 6:**
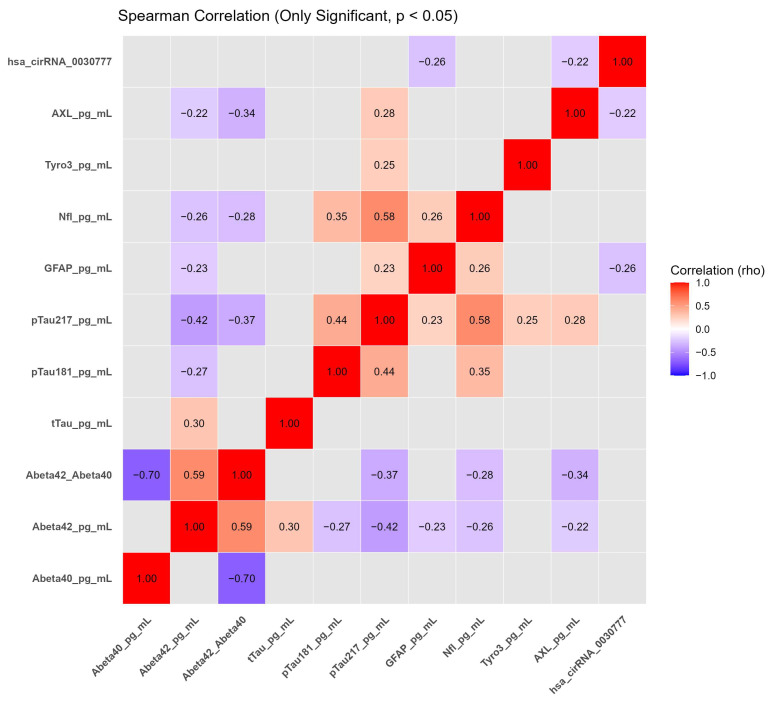
Spearman correlation matrix of plasma neurodegenerative and inflammatory biomarkers in Alzheimer’s disease (AD) patients and healthy controls (HC). Spearman rank correlation coefficients (*r*) and exact *p*-values are reported for all pairwise comparisons (see Supplementary Table S5). Correlation analyses were performed without adjustment for multiple comparisons and should therefore be interpreted as exploratory. Color intensity reflects the strength and direction of the correlations.

To explore the relationships among plasma neurodegenerative and inflammatory biomarkers, a correlation matrix was generated using Spearman’s rank correlation analysis (Figure 6). Correlation analyses were conducted in an exploratory manner, and no adjustment for multiple comparisons was applied.

The Aβ42/Aβ40 ratio showed inverse associations with GFAP (r = −0.34, *p* = 0.0292) and phosphorylated tau-217 (r = −0.32, *p* = 0.0136), consistent with the established inverse relationship between amyloid burden and tau-related pathology (Supplementary Table S5).

Positive correlations were observed between pTau181 and pTau217 (r = 0.45, *p* = 0.0001) as well as NfL (r = 0.37, *p* = 0.0036). In addition, pTau217 showed positive associations with NfL (r = 0.60, *p* < 0.0001), Tyro3 (r = 0.27, *p* = 0.0094), and AXL (r = 0.25, *p* = 0.0136), supporting the interrelated nature of tau pathology, axonal injury, and innate immune signaling in AD (Supplementary Table S5).

Circulating hsa_circ_003077 levels demonstrated weak inverse associations with AXL (r = −0.22, *p* = 0.0297) and GFAP (r = −0.26, *p* = 0.0104). These associations indicate that lower plasma hsa_circ_003077 levels tend to co-occur with increased markers of glial activation and immune signaling, without implying a direct biological or mechanistic role.

## 4. Discussion

AD is a progressive neurodegenerative disorder that ultimately leads to loss of cognitive function [29]. Early diagnosis of AD is crucial, as no effective drug treatment is currently available despite extensive research [30]. While diagnosis often relies on clinical assessment, cerebrospinal fluid (CSF) biomarkers (β-amyloid, phosphorylated tau), and advanced neuroimaging techniques, these methods are limited by invasiveness, high cost, and accessibility [31]. Thus, identifying reliable, non-invasive biomarkers is essential for early diagnosis, risk prediction, and guiding clinical management of AD.

circRNAs represent a distinct class of endogenous non-coding RNAs that exert crucial regulatory functions in gene expression [32]. Recent studies have highlighted their involvement in the pathogenesis of neurodegenerative diseases, particularly AD, where they appear to modulate the expression and accumulation of pathogenic proteins [33]. Due to their high stability, enrichment in the central nervous system, and capacity to cross the blood–brain barrier, circRNAs have emerged as promising non-invasive biomarkers for the early detection and differentiation of AD [34]. Several circRNAs have been identified in clinical studies as potential diagnostic indicators of AD [20].

This study investigated plasma-based biomarkers to differentiate AD patients from healthy controls, with a focus on classical neurodegenerative markers, glial and inflammatory mediators, TAM receptors, and a novel circRNA, hsa_circ_003077. As expected, AD patients demonstrated a significant decrease in the Aβ42/Aβ40 ratio alongside elevated levels of total Tau (tTau) and phosphorylated Tau species (pTau217). These results are consistent with the well-established amyloid and tau pathology, which leads to inflammation, synaptic impairment, neuronal loss, and thus to cognitive decline and behavioral abnormalities in AD [35] and confirm that these plasma markers remain highly relevant for disease characterization. The negative correlation between the Aβ42/Aβ40 ratio and tau markers was compatible with recent studies investigating AD-based plasma biomarkers [36].

Markers related to glial activation and axonal damage, such as GFAP and NfL, also showed disease-associated alterations. GFAP levels were significantly elevated in AD patients, indicating pronounced astrocytic activation in response to neurodegenerative processes [37,38]. In contrast, NfL levels, a robust marker of axonal injury and neurodegeneration, were significantly elevated in AD plasma. These findings are consistent with previous studies highlighting the diagnostic and prognostic value of NfL in neurodegenerative diseases [37].

TAM receptors are a family of receptor tyrosine kinases, comprising Tyro3, AXL, and MerTK (Mer Tyrosine Kinase). They help suppress excessive inflammation by clearing apoptotic cells and downregulating pro-inflammatory signals [39]. The dysregulation of TAM signaling has been implicated in pathological processes leading to neuroinflammation, myelination abnormalities, neurodegeneration, and ischemic injury to neurons [40]. Regarding the TAM receptors, neither AXL nor Tyro3 levels showed significant differences between AD patients and healthy controls. These observations suggest a potential involvement of innate immune regulatory pathways in AD-related neuroinflammation, consistent with recent studies [41,42]. Although plasma levels of Tyro3 and AXL did not significantly differ between AD patients and healthy controls, moderate associations with GFAP and pTau217 were observed. These findings suggest that alterations in innate immune regulatory pathways may accompany AD-related neuroinflammation, even in the absence of marked changes in circulating receptor concentrations. However, the lack of group-level differences indicates that plasma TAM receptor levels alone may have limited diagnostic utility.

A central exploratory objective of this study was the evaluation of the circular RNA hsa_circ_003077 in plasma. A notable finding was the significantly reduced expression of hsa_circ_003077 in AD patients compared with healthy controls. Circulating levels of hsa_circ_003077 showed weak-to-moderate inverse associations with selected markers of glial activation and innate immune signaling, including GFAP and AXL. These observations indicate that lower plasma hsa_circ_003077 levels tend to co-occur with increased markers of neurodegeneration-associated processes in AD. Importantly, these associations are correlative in nature and do not imply a direct biological or functional role for hsa_circ_003077 in disease pathogenesis. Plasma circRNA measurements represent systemic circulating signatures and may not directly reflect tissue-specific expression or molecular activity within the central nervous system.

A limitation of the present study is the absence of functional or bioinformatic analyses to directly address the molecular mechanisms underlying the observed association between hsa_circ_003077 and Alzheimer’s disease–related biomarkers. Accordingly, the biological significance of hsa_circ_003077 remains speculative. Previous studies have suggested that circRNAs may influence neurodegenerative processes through mechanisms such as miRNA sponging, regulation of target gene expression, and modulation of pathways involved in amyloid processing, tau phosphorylation, and neuroinflammation [13,43]. In this context, future investigations integrating bioinformatic prediction approaches and experimental validation may help to further elucidate whether hsa_circ_003077 is involved in such regulatory networks in AD.

ROC curve analysis indicated that hsa_circ_003077 exhibited the highest diagnostic performance among the evaluated plasma biomarkers in this cohort (AUC = 0.90), suggesting its potential as a disease-associated circulating marker for distinguishing AD patients from healthy controls. However, this finding should be interpreted with caution, as the study was exploratory in nature, involved a relatively modest sample size, and lacked external validation. These factors may limit statistical power and increase the risk of overestimating diagnostic performance metrics. In addition, all analyses were conducted within a single cohort, restricting the generalizability of the results, and the absence of functional or bioinformatic analyses precludes mechanistic interpretation of the observed circRNA associations. Accordingly, further investigations in larger, independent, and ideally multicenter cohorts, incorporating longitudinal designs and integrative mechanistic approaches, will be required to confirm the diagnostic value and biological relevance of hsa_circ_003077 in Alzheimer’s disease.

## 5. Conclusions

In conclusion, the findings from this study not only reaffirm the diagnostic value of classical AD biomarkers but also introduce hsa_circ_003077 as a highly promising candidate for early and accurate detection of AD. The combination of tau pathology, axonal damage, immune signaling, and circRNA dynamics offers a multidimensional view of AD pathogenesis and supports the integration of novel molecular tools in clinical practice. Additionally, combining circRNAs with other biomarkers and imaging tools can effectively enhance diagnostic capabilities. Further validation in larger, longitudinal cohorts is warranted to confirm the clinical applicability.

## Figures and Tables

**Table 1:** Demographic Characteristics of Healthy Controls (HC) and Alzheimer’s Disease (AD) Patients.

Parameters	HC	AD
*N*	25	69
Sex	F = 12, M = 13	F = 32, M = 37
Age years (mean ± SD)	61 ± 3	63 ± 4
MMSE (mean ± SD)	29 ± 1.3	19 ± 5.3
CSF tTau (pg/mL) (median [IQR])	138 [203–150]	557 [455–1066] ^a^
CSF pTau181 (pg/mL) (median [IQR])	43 [56–31]	81 [44–115] ^a^
CSF Aβ_42_ (pg/mL) (median [IQR])	1325 [1430–775]	697 [510–821] ^a^
Plasma Abeta40 pg/mL (median [IQR])	163 [188–403]	143 [103–589] ^a^
Plasma Abeta42 pg/mL (median [IQR])	123 [426–1005]	334 [351–1077] ^ns^
Plasma tTau pg/mL (median [IQR])	77 [213–558]	85 [206–596] ^a^
Plasma pTau181 pg/mL (median [IQR])	4 [2–9]	4 [3–16] ^ns^
Plasma pTau217 pg/mL (median [IQR])	0.4 [0.2–1.4]	1.4 [0.3–3.0] ^a^
Plasma GFAP pg/mL (median [IQR])	22 [31–93]	156 [191–227] ^a^
Plasma NfL pg/mL (median [IQR])	3 [15–30]	28 [11–90] ^a^
Plasma Tyro3 pg/mL (median [IQR])	6 [19–44]	21 [10–96] ^ns^
Plasma AXL pg/mL (median [IQR])	4 [29–68]	12 [24–85] ^ns^
Plasma hsa_circ_0030777 2^–ΔCt (median [IQR])	0.8 [1.1–3.1]	0.4 [0.5–1.6] ^a^

Data are presented as mean ± standard deviation (SD) for normally distributed variables (age and MMSE scores) and as median with interquartile range [IQR] for non-normally distributed CSF and plasma biomarkers. Statistical comparisons between HC and AD groups were performed using Student’s t-test for normally distributed variables (age and MMSE scores) and the Mann–Whitney U test for non-normally distributed CSF and plasma biomarkers. Superscript “a” indicates a statistically significant difference compared with the HC group (*p* < 0.05), while “ns” denotes a non-significant difference.

## Data Availability

The datasets generated and/or analyzed during the current study are available from the corresponding author upon reasonable request.

## Supplementary Materials

The following supporting information can be downloaded at: https://www.mdpi.com/article/10.3390/biom16030356/s1, Table S1: Genomic annotation and database identifiers of hsa_circ_0030777; Table S2: Comparison of plasma biomarker levels between cognitively healthy controls (HC) and Alzheimer's disease (AD) participants; Table S3: Diagnostic performance of plasma biomarkers for Alzheimer’s disease: area under the ROC curve (AUC) and 95% confidence intervals (CI); Table S4: Diagnostic performance of plasma biomarkers for distinguishing Alzheimer's disease (AD) from cognitively healthy controls (HC) based on ROC curve analysis; Table S5: Spearman rank correlation coefficients between plasma biomarkers.
