# Supplementary material for: Evaluation of Plasma-Derived hsa_circ_003077 for Non-Invasive Diagnosis of Alzheimer’s Disease
Source: Biomolecules. 2026 Feb 26;16(3):356. doi: 10.3390/biom16030356 (PMC13023876; doi:10.3390/biom16030356)
Supplement: Supplementary file 1 [file biomolecules-16-00356-s001.zip › Biomolecules Supplementary/Supplementary Table S1.pdf]

Supplementary Table 1. Genomic annotation and database identifiers of hsa_circ_0030777

circRNA name	Host Gene	Chromosome	Start	End	Strand	Genome build	circBase ID	circAtlas ID
hsa_circ_0030777	PCCA	chr13	101077886	101101559	+	hg19	hsa_circ_0030777	hsa-PCCA_0009

Genomic coordinates and host gene information were obtained from circBase (hg19/GRCh37). circBase annotations are based on the hg19 genome assembly, whereas circAtlas annotations, when available, were retrieved using the GRCh38/hg38 reference genome.
